# Impaired neurodevelopmental pathways in autism spectrum disorder: a review of signaling mechanisms and crosstalk

**DOI:** 10.1186/s11689-019-9268-y

**Published:** 2019-06-15

**Authors:** Santosh Kumar, Kurt Reynolds, Yu Ji, Ran Gu, Sunil Rai, Chengji J. Zhou

**Affiliations:** Department of Biochemistry and Molecular Medicine, Institute for Pediatric Regenerative Medicine of Shriners Hospitals for Children, University of California at Davis School of Medicine, 2425 Stockton Blvd, Sacramento, CA 95817 USA

**Keywords:** WNT, BMP/TGF-β, SHH, FGF, Retinoic acid (RA), Signaling crosstalk, Autism spectrum disorder, Neurodevelopmental disorders

## Abstract

**Background:**

The development of an autistic brain is a highly complex process as evident from the involvement of various genetic and non-genetic factors in the etiology of the autism spectrum disorder (ASD). Despite being a multifactorial neurodevelopmental disorder, autistic patients display a few key characteristics, such as the impaired social interactions and elevated repetitive behaviors, suggesting the perturbation of specific neuronal circuits resulted from abnormal signaling pathways during brain development in ASD. A comprehensive review for autistic signaling mechanisms and interactions may provide a better understanding of ASD etiology and treatment.

**Main body:**

Recent studies on genetic models and ASD patients with several different mutated genes revealed the dysregulation of several key signaling pathways, such as WNT, BMP, SHH, and retinoic acid (RA) signaling. Although no direct evidence of dysfunctional FGF or TGF-β signaling in ASD has been reported so far, a few examples of indirect evidence can be found. This review article summarizes how various genetic and non-genetic factors which have been reported contributing to ASD interact with WNT, BMP/TGF-β, SHH, FGF, and RA signaling pathways. The autism-associated gene ubiquitin-protein ligase E3A (*UBE3A*) has been reported to influence WNT, BMP, and RA signaling pathways, suggesting crosstalk between various signaling pathways during autistic brain development. Finally, the article comments on what further studies could be performed to gain deeper insights into the understanding of perturbed signaling pathways in the etiology of ASD.

**Conclusion:**

The understanding of mechanisms behind various signaling pathways in the etiology of ASD may help to facilitate the identification of potential therapeutic targets and design of new treatment methods.

## Background

Autism spectrum disorder (ASD) is a multifactorial neurodevelopmental disorder characterized by impaired social interactions and elevated repetitive behaviors, in which various circuits in the sensory, prefrontal, hippocampal, cerebellar, striatal, and other midbrain regions are perturbed [1, 2]. Compared to de novo non-coding variations, the de novo coding variants have a strong association with ASD as shown by the whole-genome sequence association (WGSA) studies in 519 ASD families [3]. Kosmicki et al. further showed *CHD8*, *ARID1B*, *DYRK1A*, *SYNGAP1*, *ADNP*, *ANK2*, *DSCAM*, *SCN2A*, *ASH1L*, *CHD2*, *KDM5B*, and *POGZ* genes with ≥ 3 class 1 de novo protein-truncating variants (PTVs) in individuals with ASD [4]. *ANK2*, *CHD8*, *CUL3*, *DYRK1A*, *GRIN2B*, *KATNAL2*, *POGZ*, *SCN2A*, and *TBR1* genes are identified as high-confidence ASD risk genes, of which *CHD8* is strongly associated with ASD because of the largest number of de novo loss-of-function (LOF) mutations observed in patients [5, 6]. Various gene mutations reported in ASD patients are either core components of the WNT signaling pathway or their modulators [7–9]. More recently, a few reports support the idea of modulation of bone morphogenetic protein (BMP) signaling as a contributing factor in ASD model organisms and humans. For instance, Neuroligins (*NLGN*), fragile X mental retardation 1 (*FMR1*), ubiquitin-protein ligase E3A (*UBE3A)*, and *DLX*, which modulate BMP signaling, have been found to be associated with ASD [10–13]. Dysregulation of sonic hedgehog (SHH) [14, 15], fibroblast growth factor (FGF) [16], transforming growth factor β (TGF-β) [17, 18], and retinoic acid (RA) [19] signaling pathways have also been implicated in the pathogenesis of ASD. Valproic acid (VPA), used in the treatment of epilepsy and bipolar disorder, may affect the WNT/β-catenin signaling pathway. However, its prenatal exposure in rats also causes susceptibility to autism [20]. Due to the scarcity of information on the mechanisms underlying the etiologies of ASD, the success of therapeutic strategies is greatly limited.

## Altered WNT signaling in ASD

WNT signaling is fundamental for neurodevelopmental and post-neurodevelopmental processes, such as CNS regionalization, neural progenitor differentiation, neuronal migration, axon guidance, dendrite development, synaptogenesis, adult neurogenesis, and neural plasticity [21–30], and therefore, any perturbation in WNT signaling may trigger the advent of disorders related to the structures and functions of the CNS [9, 31]. Studies in genetically modified animal models and human induced pluripotent stem cell (hiPSC) models, along with large-scale human genomic studies in several neurodevelopmental disorders over the last few decades, have revealed the importance of spatiotemporal regulation of WNT signaling throughout the lifespan of an animal [9]. Moreover, dysregulation of WNT signaling has been reported in several psychiatric disorders, including ASD, bipolar disorder, and schizophrenia, as well as in case of intellectual disability [8, 9, 31–34]. Although several genetic and epigenetic factors have been linked with the etiologies of neurodevelopmental disorders, they often seem to affect a few common processes, such as chromatin remodeling, WNT signaling, and synaptic function [32, 35, 36]. Despite the heterogeneity in WNT signaling, it is broadly classified into “canonical” (β-catenin-dependent) and “non-canonical” (β-catenin-independent) pathways [37, 38]. Both canonical and non-canonical WNT signaling pathways play crucial roles in neural development and related neurodevelopmental disorders.

### Genetic etiologies

**Fig. 1:**
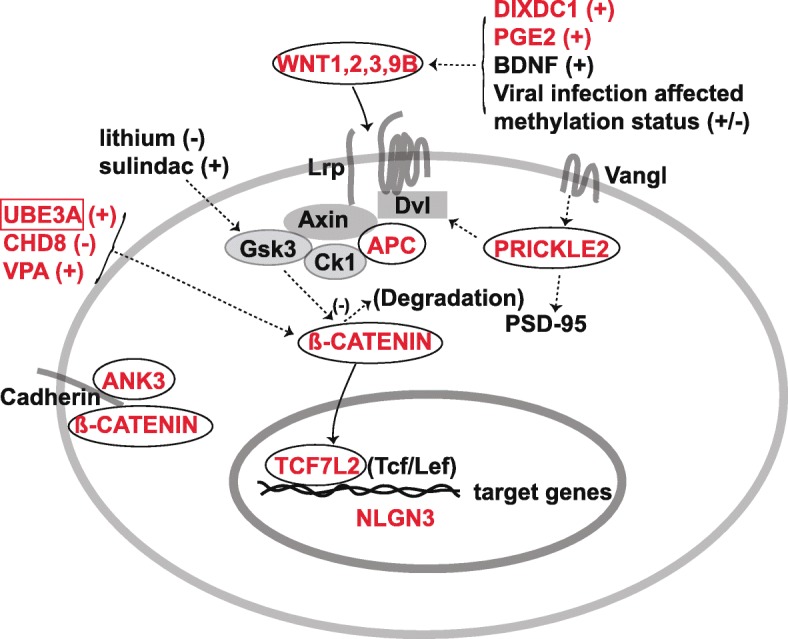
Possible interactions between ASD causal genes and WNT signaling. Most molecules (red) encoded by the ASD-associated genes are either core components of WNT signaling pathways, such as WNTs, APC, β-catenin, TCF7L2, and PRICKLE2, or their modulators, such as DIXDC1, PGE2, UBE3A, and CHD8. ANK3 interacts with β-catenin at the plasma membrane. Note: plus sign indicates upregulation; minus sign indicates downregulation

Several genetic loci/mutations linked to and/or reported in ASD patients are either core components of canonical WNT signaling, such as β-catenin (*CTNNB1*) [8, 9, 36, 39] and adenomatous polyposis coli (*APC*) [1], or non-canonical WNT signaling, such as *PRICKLE2* [40], suggesting crucial roles of both canonical and non-canonical WNT signaling pathways in the etiologies of ASD (Table 1 and Fig. 1).

**Table 1:** Autism spectrum disorder (ASD) causal genes influencing WNT, BMP/TGF-β, SHH, FGF, and RA signaling pathways in vertebrates and invertebrates

ASD causal genes	Region/neurons/cells in which gene function is affected	Species	Affected signaling pathway	Phenotypes/downstream targets	Citations
*5-HT* GOF (gain-of-function)	Blood	Human	TGF-β	TGF-β pathway identified as a novel hyperserotonemia-related ASD genes	[18]
*ALDH1A3*		Human	RA		[135]
*ANK3* LOF (loss-of-function)	P19 cells, proliferating neural progenitors of E16 mouse cortices, E15 brain slices	Mouse	WNT (canonical)	Increases proliferation of neural progenitor cells and nuclear β-catenin	[85]
*APC* LOF	Forebrain neurons, and hippocampal, cortical, and striatal regions	Mouse	WNT (canonical)	Learning and memory impairments and autistic-like behaviors (increased repetitive behaviors, reduced social interest)	[1]
BTBR T^+^ Itpr3^tf^/J (BTBR) mice	Spleen and brain tissues	Mouse	TGF-β	Decreased TGF-β levels	[17]
*CD38*	Lymphoblastoid cell lines	Human and mouse	RA	Upregulation of CD38 by RA	[140, 141]
*CHD8*^−/−^ LOF	Whole	Mouse	WNT (canonical)	Embryonically lethal	[87]
*CHD8*^+/−^ LOF	Nucleus accumbens (NAc)	Mouse	WNT (canonical)	Macrocephaly, craniofacial abnormalities, and behavioral deficits; WNT signaling upregulates in the nucleus accumbens (NAc) region of the brain	[89]
*CTNNB1* LOF	Parvalbumin interneurons	Mouse	WNT (canonical)	Impaired object recognition and social interactions; elevated repetitive behaviors; enhanced spatial memory	[66]
*CTNNB1* cKO LOF	Dorsal neural folds	Mouse	WNT (canonical)	Spina bifida aperta, caudal axis bending, and tail truncation	[65]
*CTNNB1* haploinsufficiency LOF	Whole	Human and mouse	WNT (canonical)	Neuronal loss, craniofacial anomalies, and hair follicle defects	[64]
*DHCR7* LOF	MEFs	Mouse	SHH	Impaired SMO and reduced SHH signaling	[111]
*DIXDC1* LOF	Mouse cortex	Human and mouse	WNT (canonical)	Impaired dendrite and spine growth, positive modulator of WNT signaling	[79]
*Dlx5* GOF	2B1 cell line	Mouse	BMP	Upregulation of BMP binding endothelial regulator (Bmper)	[13]
*DNlg4* LOF	NMJ	Drosophila	BMP	Reduced growth of neuromuscular junctions (NMJs) with fewer synaptic boutons	[10]
*EN2* GOF	Post-mortem samples	Human	SHH	Elevated SHH expression	[117]
*FGF22*/*FGF7* LOF	Hippocampal CA3 pyramidal neurons	Mouse	FGF	Impaired synapse formation	[124]
FMRP depletion	COS-7 cells	Monkey	BMP	Increase in BMPR2 and activation of LIMK1, stimulates reorganization of actin to promote neurite outgrowth and synapse formation	[11]
*FOXN1*		Human	RA		[135]
*mGluR5* LOF	Cortical neurons	Mouse	FGF	Increased NGF and FGF10 mRNA levels	[125]
*PGE2*	Differentiating neuronal cells	Humans	WNT (canonical)	Upregulation of *WNT3* and *TCF4*	[80, 93, 94]
*PRICKLE2* LOF	Hippocampal neurons	Mouse	WNT (non-canonical)	Altered social interaction, learning abnormalities, and behavioral inflexibility	[40]
*PTCHD1* LOF	Dentate gyrus	Mouse	SHH (hypothetical)	SHH independent; disrupted synaptic transmission	[109]
*RERE*		Human	RA		[137]
*RORA* LOF	Lymphoblastoid cell lines	Human	RA	Reduced protein levels of RORA and BCL-2 in autistic brain; aberrant methylation	[133]
*TCF7L2* LOF		Human and mouse	WNT (canonical)	Required for thalamocortical axonal projection formation	[68–71]
*UBE3A* GOF	Prefrontal cortex	Mouse	RA	Negative regulation of ALDH1A2; impaired RA-mediated synaptic plasticity	[139]
*ube3a* LOF	NMJ	Drosophila	BMP	Compromised endocytosis in the NMJs and an upregulated BMP signaling in the nervous system	[12]
*UBE3A*^T485A^ LOF	HEK293T cells	Human	WNT (canonical)	Stabilizes nuclear β-catenin and stimulates canonical WNT signaling	[78]
*WNT1*, *WNT2*, *WNT3*, *WNT9B*		Human	WNT (canonical)	Elevated *WNT3* expression in the prefrontal cortex of ASD patients	[34, 41–45]

#### Core components of canonical WNT signaling

##### WNTs

Among 19 *WNT* ligands, mutations in *WNT1*, *WNT2*, *WNT3*, and *WNT9B* have been linked with ASD. A rare *WNT1* missense variant found in ASD patients has a higher capability than the wild-type *WNT1* to activate WNT/β-catenin signaling [34]. Rare variants in *WNT2*, *WNT3*, and *WNT9B* have also been found in ASD patients [41–44]. Notably, *WNT3* expression is elevated in the prefrontal cortex of ASD patients [45], suggesting overactivation of WNT signaling leads to ASD pathogenesis. In animal models, *Wnt1* is required for midbrain and cerebellar development [46–48]. *Wnt2* has been shown to be sufficient for cortical dendrite growth and dendritic spine formation, and its expression is regulated by a brain-derived neurotrophic factor (BDNF) [49], while altered dendritic spines result in neurodevelopmental and neurodegenerative disorders [49]. *Wnt3* is essential for gastrulation and regulates hippocampal neurogenesis [50, 51]. Loss-of-function of *T-brain-1* (*Tbr1*), a T-box transcription factor and one of the high-confidence ASD risk genes, in cortical layer 6 neurons (*Tbr1*^layer6^ mutant) during late mouse gestation has been reported to cause a decrease in inhibitory synaptic density and excitatory synapse numbers [52]. It is important to note that the restoration of *Wnt7b* expression rescues the synaptic deficit in *Tbr1*^layer6^ mutant neurons [52]. *Wnt9b* promotes lip/palate formation and fusion [53, 54], while its role in neurodevelopment remains unclear. In addition to the other WNT ligands, there are various types of WNT receptors, including FZD1 to FZD10, LRP5/6, RYK, and ROR1/2 [30, 55], whose roles in ASD etiology remain elusive.

##### APC

The tumor suppressor APC is a key component of the β-catenin-destruction complex [56]. Human *APC* inactivating gene mutations have been linked to ASD [57, 58]. Compared with wild-type littermates, conditional knockout (cKO) *Apc* mice exhibit learning and memory impairment and autistic-like behaviors [1]. β-catenin and canonical WNT target gene expressions (*Dkk1*, *Sp5*, *Neurog1*, *Syn2*) are increased in *Apc-*cKO forebrain neurons [1]. Moreover, the lysates from the hippocampal, cortical, and striatal regions of *Apc-*cKO mice showed higher β-catenin levels compared to those of control mice [1]. These results also indicate that overactivation of WNT/β-catenin signaling may be a cause of ASD.

##### CTNNB1 (β-catenin)

β-Catenin is a key intracellular molecule in the canonical WNT signaling pathway and plays significant roles in development and disease [59, 60]. De novo *CTNNB1* mutations have been reported in individuals with ASD, intellectual disability, microcephaly, motor delay, and speech impairment [36, 39, 61–63]. *CTNNB1* haploinsufficiency has been found to cause neuronal loss, craniofacial anomalies, and hair follicle defects in both humans and mice [64]. Conditional ablation of β-catenin in the dorsal neural folds of mouse embryos represses the expression of *Pax3* and *Cdx2* at the dorsal posterior neuropore and leads to a decreased expression of the WNT/β-catenin signaling target genes *T*, *Tbx6*, and *Fgf8* at the tail bud, resulting in spina bifida aperta, caudal axis bending, and tail truncation [65]. Conditional ablation of β-catenin in parvalbumin interneurons in mice leads to impaired object recognition and social interactions, as well as elevated repetitive behaviors, which are core symptoms of ASD patients, and surprisingly, they showed enhanced spatial memory [66]. These mice have reduced c-Fos activity in the cortex, which is unaffected in the dentate gyrus and the amygdala, suggesting a cell type-specific role of β-catenin in the regulation of cognitive and autistic-like behaviors [66].

##### TCF7L2 (TCF4)

TCF7L2 is one of the TCF/LEF1 transcription factors in the canonical WNT/β-catenin signaling pathway and is associated with type II diabetes in humans [67]. De novo loss-of-function variants of *TCF7L2* have been found in ASD patients [68, 69]. In mice, Tcf7l2 is required for the formation of thalamocortical axonal projections, as is the key Wnt co-receptor Lrp6 [70, 71], suggesting that abnormal thalamocortical axonal inputs may contribute to ASD. It remains unclear if other members of the TCF/Lef1 transcription factors are associated with ASD.

#### Core components of non-canonical WNT signaling

##### PRICKLE2

*PRICKLE2* variants (p.E8Q and p.V153I) have been reported in ASD patients [40]. *PRICKLE2*’s role in ASD is further supported by the finding of a 3p interstitial deletion including *PRICKLE2* in identical twins with ASD [72]. *Prickle2*-deficient mice display ASD-like phenotypes, such as altered social interaction, learning abnormalities, and behavioral inflexibility [40]. PRICKLE2 is known to interact with post-synaptic density protein-95 (PSD-95), and this relationship is enhanced by Vangl2, a key component in the non-canonical WNT/PCP (planar cell polarity) pathway [73]. Reduced dendrite branching, synapse number, and PSD size have been observed in hippocampal neurons of *Prickle2*-deficient mice [40]. An in vitro study shows that Prickle1 and Prickle2 promote neurite outgrowth via a Dvl-dependent mechanism [74]. Future works need to address the involvement of other PCP genes and the signaling interaction between the PCP and WNT/β-catenin signaling pathways in ASD etiology.

#### Modulators and effectors of WNT signaling in ASD etiology

Several ASD-associated genes are direct or indirect modulators of WNT signaling, such as ankyrin-G (*ANK3*) [75–77], chromodomain helicase DNA-binding protein 8 (*CHD8*) [5, 6], HECT domain E3 ubiquitin ligase (*UBE3A*) [78], DIX domain-containing 1 (*DIXDC1*) [79], and Prostaglandin E2 (*PGE2*) [80] (Table 1 and Fig. 1). Intriguingly, a recent study suggests that the ASD-associated gene Neuroligin 3 (Nlgn3) is a direct downstream target of WNT/β-catenin signaling during synaptogenesis [81] (Fig. 1).

##### ANK3

Whole-genome and whole-exome sequencing studies in ASD patients have identified mutations in *ANK3* gene [75–77]. Ankyrin-G, a scaffolding protein encoded by *ANK3* gene, localizes to the axon initial segment (AIS) and the nodes of Ranvier, where it has roles in the assembly and maintenance of the AIS and neuronal polarity [82, 83]. Ankyrin-G facilitates cell-cell contact by binding to E-cadherin at a conserved site distinct from that of β-catenin and localizes it to the cell adhesion site along with β-2-spectrin in early embryos and cultured epithelial cells [84]. Ankyrin-G is enriched at the ventricular zone of the embryonic brain, where it regulates the proliferation of neural progenitor cells [85]. Ankyrin-G LOF increases the proliferation of neural progenitor cells and nuclear β-catenin, probably by disruption of the β-catenin/cadherin interaction [85].

##### CHD8

CHD8, an ATP-dependent chromatin remodeler, interacts with β-catenin and negatively regulates the expression of β-catenin-targeted genes [86]. CHD8 binding to p53 leads to the formation of a trimeric complex with histone H1 on chromatin, which suppresses p53-dependent transactivation and apoptosis during early embryogenesis [87]. CHD8 is also required for the expression of E2 adenovirus promoter-binding factor target genes during the G1/S transition of the cell cycle [88]. *Chd8* gene knockout (*Chd8*^−/−^) in mice is embryonic lethal [87], whereas its heterozygous LOF mutations (*Chd8*^+/−^) result in mice with macrocephaly, craniofacial abnormalities, and behavioral deficits [89]. Its knockdown in SK-N-SH human neural progenitor cells alters the expression of genes involved in neuronal development [90]. WNT signaling is upregulated in the nucleus accumbens (NAc) region of the brain of *Chd8*^+/−^ mice, highlighting the critical role CHD8 plays in the regulation of WNT signaling in the NAc [89].

##### UBE3A

Dysfunction of *UBE3A* is linked to autism, Angelman syndrome, and cancer [78]. *UBE3A*^T485A^, a de novo autism-linked *UBE3A* mutant that disrupts phosphorylative control of UBE3A activity, ubiquitinates multiple proteasome subunits, reduces proteasome subunit abundance and activity, stabilizes nuclear β-catenin, and stimulates canonical WNT signaling more effectively than the wild-type UBE3A [78].

##### DIXDC1

Rare missense variants in *DIXDC1* were identified in ASD patients [79]. These variants prevent phosphorylation of DIXDC1 isoform 1, causing impairment to dendrite and spine growth [79]. DIXDC1 is a positive modulator for WNT signaling and regulates excitatory neuron dendrite development and synapse function in the mouse cortex [79]. MARK1, which is also linked to ASD, phosphorylates DIXDC1 to regulate dendrite and spine development through modulation of the cytoskeletal network in an isoform-specific manner [79]. Dixdc1-deficient mice exhibit behavioral disorders, including reduced social interaction, which can be alleviated through pharmacological inhibition of Gsk3 to upregulate WNT/β-catenin signaling [91, 92]. These studies suggest a potential approach to ASD treatment through manipulation of WNT/β-catenin signaling activities.

##### PGE2

PGE2, an endogenous lipid molecule, has been linked to ASD and alters the expression of downstream WNT-regulated genes previously associated with neurodevelopmental disorders [80]. The link between prostaglandin and autism came from the report of Möbius sequence with autism and positive history of misoprostol use during pregnancy [93]. The prostaglandin analog misoprostol is used as an abortifacient as well as for the prevention of gastric ulcers. Among seven children with ASD, four (57.1%) had prenatal exposure to misoprostol [93, 94]. In undifferentiated stem cells, PGE2 downregulates *PTGS2* expression and upregulates *MMP9* and *CCND1* expression, whereas in differentiating neuronal cells, PGE2 causes upregulation of *WNT3*, *TCF4*, and *CCND1* [80].

##### NLGN3

Mutations in neuroligins *NLGN3* and *NLGN4* have been reported in autistic patients [95]. These type I transmembrane proteins are neural cell adhesion molecules and are required for the formation and development of synapses [10]. Chromatin immunoprecipitation and promoter luciferase assays demonstrate that WNT/β-catenin signaling directly regulates Nlgn3 expression [81]. It will be important to address whether WNT/β-catenin signaling regulates other ASD-associated genes.

## Altered TGF-β/BMP signaling in ASD

The TGF-β/activin and the bone morphogenetic protein (BMP)/growth and differentiation factor (GDF) are the two subgroups of TGF-β superfamily [96]. BMPs constitute the largest subdivision of the TGF-β superfamily [97] and are critical in the development of the nervous system [98]. Their signaling has been shown to be dysregulated in ASD. BMPs regulate the expression of various genes by the canonical pathway (Smad-dependent) and non-canonical pathways (such as MAPK cascade) [99]. In the canonical pathway, the binding of BMPs to type I or type II serine/threonine kinase receptors forms a heterotetrameric complex. This leads to the transphosphorylation of the type I receptor by the type II receptor. The type I receptor then phosphorylates the R-Smads (Smad1/5/8). The phosphorylated Smad1/5/8 along with the co-Smad (Smad4) translocate to the nucleus and regulate gene expression. There are various factors such as plasma membrane co-receptors and extracellular and intracellular factors known to modulate BMP signaling [99]. BTBR T^+^Itpr3^tf^/J (BTBR) mice are widely used in the study of ASD [17]. It has been reported that TGF-β levels are reduced in BTBR mice in comparison with B6 mice [17] (Table 1). Significant changes in the expression of TGF-β have been found in the spleen and brain tissues of BTBR mice compared to those in adenosine A2A receptor (A2AR) agonist CGS 21680 (CGS)-treated mice [17]. ASD has been linked with higher levels of serotonin (5-hydroxytryptamine or 5-HT) in the blood [18] (Table 1). In a network-based gene set enrichment analysis (NGSEA), components of the TGF-β pathway have been identified as novel hyperserotonemia-related ASD genes, based on LOF and missense de novo variants (DNVs) [18].

### NLGN4

Drosophila neuroligin 4 (*DNlg4*) LOF results in reduced growth of neuromuscular junctions (NMJs), with fewer synaptic boutons due to the reduction in the bone morphogenetic protein (BMP) type I receptor thickvein (*Tkv*) [10], suggesting important roles of BMP signaling in normal and autistic brains.

### FMR1

Fragile X syndrome (FXS) is the most common heritable form of intellectual disability and ASD, which is caused due to the silencing of *FMR1* [11]. FMR1 protein (FMRP) depletion results in an increase in the bone morphogenetic protein type II receptor (*BMPR2*) and activation of a non-canonical BMP signaling component LIM domain kinase 1 (*LIMK1*), which stimulates reorganization of actin to promote neurite outgrowth and synapse formation [11]. Increased *BMPR2* and *LIMK1* activity has been reported in the prefrontal cortex of FXS patients compared with that of healthy subjects [11].

### UBE3A

The inhibition of BMP signaling by Ube3a has been reported to play a role in the regulation of synapse growth and endocytosis [12]. A direct substrate of ube3a, the BMP receptor Tkv, is degraded through the ubiquitin-proteasome pathway [12]. Drosophila ube3a has been known to regulate the NMJ development in the presynaptic neurons through the BMP signaling pathway [12]. Drosophila ube3a mutants have been shown viable and fertile. However, they display compromised endocytosis in the NMJs and upregulated BMP signaling in the nervous system due to an increase in *Tkv* [12].

### DLX

The *DLX* genes encoding homeodomain transcription factors have been associated with ASD [100–102]. These genes control craniofacial patterning and differentiation and survival of forebrain inhibitory neurons [100]. The BMP-binding endothelial regulator (*Bmper*) has been found upregulated in a cell line overexpressing *Dlx5* [13], suggesting dysregulated *DLX* function in ASD patients may lead to altered BMP signaling.

## Altered SHH signaling in ASD

**Fig. 2:**
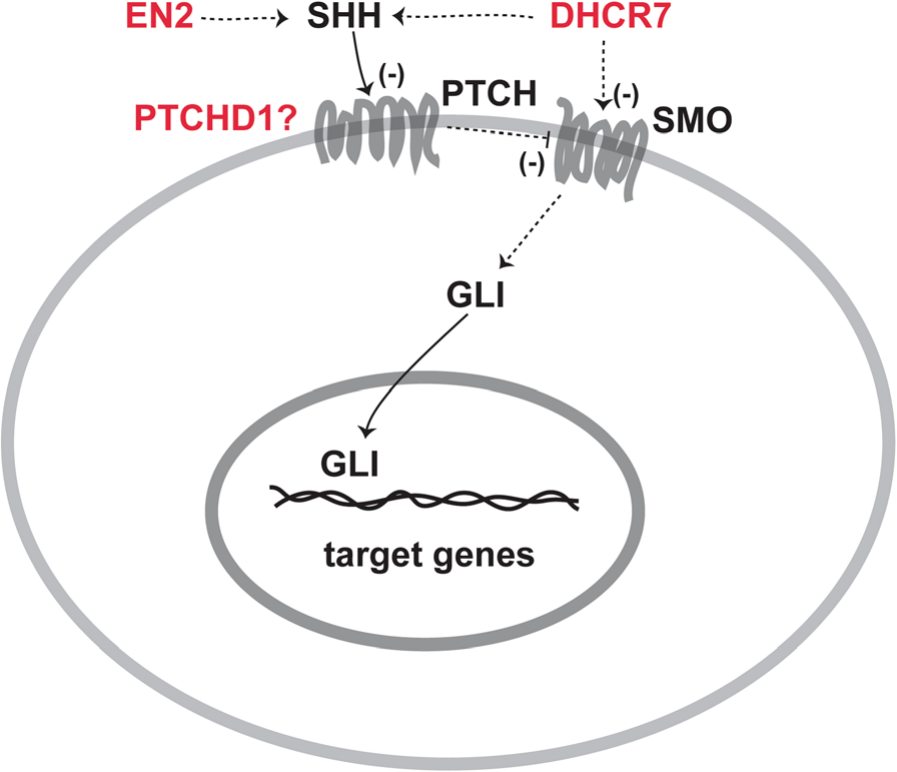
Possible interactions between ASD causal genes and SHH signaling. The genes encoded for PTCHD1, EN2, and DHCR7 are potential ASD genes. Note: minus sign indicates downregulation; question mark indicates undefined role of PTCHD1 in SHH signaling

SHH plays a crucial role in the organization of the vertebrate brain [103]. SHH has a wide range of roles in developing as well as the adult brain and drives proliferation, specification, and axonal targeting within the forebrain, hindbrain, and spinal cord [104]. Although the role of neural primary cilia in embryonic CNS patterning is well studied, their role in adult CNS plasticity has recently emerged [105]. SHH signaling at the primary cilium has been described [106] and is summarized in Fig. 2. In the absence of SHH activity, PTCH represses SMO. This leads to the phosphorylation of GLI proteins, and their subsequent proteolytic truncation into repressor forms that inhibit transcriptional activity. However, the binding of SHH to PTCH causes its internalization followed by degradation which in turn leads to SMO accumulation and phosphorylation. In this case, GLI is transported to the cytosol and enters the nucleus in its full form, which further activates target transcription. Pathological roles of SHH, Indian hedgehog (IHH), and BDNF have been suggested in children with ASD [14]. SHH signaling influences neurogenesis and neural patterning during the development of the central nervous system. Dysregulation of SHH signaling in the brain leads to neurological disorders like ASD [15]. SHH has also been associated with oxidative stress in autism [107]. Significantly higher levels of oxygen free radicals (OFR) and serum SHH protein have been demonstrated in autistic children, suggesting a pathological role of oxidative stress and SHH in ASD [108]. Figure 2 summarizes the interaction between ASD causal genes and SHH signaling.

### PTCHD1

Mutations in the gene patched domain-containing 1 (*PTCHD1*) have been reported in ASD and ID patients [109]. *Ptchd1* KO male mice exhibit cognitive alterations [109]. LOF experiments do not support a role for PTCHD1 protein in SHH-dependent signaling but reveal a disruption of synaptic transmission in the mouse dentate gyrus [109]. PTCHD1 has been shown to bind with the post-synaptic proteins PSD95 and SAP102 [110]. Ptchd1 deficiency in male mice (*Ptchd1*^*−/y*^) induces global changes in synaptic gene expression, affects the expression of the immediate-early expression genes *Egr1* and *Npas4*, and impairs excitatory synaptic structure and neuronal excitatory activity in the hippocampus, leading to cognitive dysfunction, motor disabilities, and hyperactivity [110].

### DHCR7

The impaired function of the cholesterol biosynthetic enzyme 7-dehydrocholesterol reductase (*DHCR7*) has been associated with the ASD [111]. The activation of the transmembrane protein Smoothened (SMO), through which SHH signaling is transduced, and its localization to the primary cilium is affected by conditions of reduced cholesterol biosynthesis [111].

### EN2

The transcription factor engrailed2 (EN2) has been associated with ASD [112–116]. The increased levels of *EN2* in affected individuals with *EN2* ASD-associated haplotype (rs1861972-rs1861973 A-C) further support the susceptibility of *EN2* gene for ASD [77, 117, 118]. The increased *EN2* levels result in the elevated levels of the *SHH* expression as reported in post-mortem samples [117]. *SHH* is one of the genes flanking *EN2* which is coexpressed during brain development [119, 120].

## Altered FGF signaling in ASD

FGF signaling plays a crucial role in brain patterning, and its malfunction can result in various neurological disorders [121]. There are 18 secreted FGFs and 4 tyrosine kinase FGF receptors (FGFRs) reported in the mammalian FGF family whose interaction is regulated by cofactors and extracellular binding proteins [122]. Activation of FGFRs leads to the phosphorylation of tyrosine residues which further results in the interaction between cytosolic adaptor proteins and the RAS-MAPK, PI3K-AKT, PLCγ, and STAT intracellular signaling pathways [122]. Dysregulation of FGF signaling has been suggested to play a role in the pathogenesis of ASD [16]. For instance, cortical abnormalities observed in autistic brains have been associated with defective FGF signaling [121, 123]. Perturbations in the number of excitatory and inhibitory synapses have been implicated in ASD [124]. Mutant mice lacking FGF22 or FGF7, which displayed impaired synapse formation in the hippocampal CA3 pyramidal neurons, have been reported [124], supporting the pathological role of dysregulated FGF signaling in ASD (Table 1). The metabotropic glutamate receptor 5 (*mGluR5*) LOF results in aberrant dendritogenesis, one of the characteristics observed in autistic brains, in the cortical neurons by increasing nerve growth factor (NGF) and FGF10 mRNA levels [125] (Table 1).

## Altered retinoic acid signaling in ASD

**Fig. 3:**
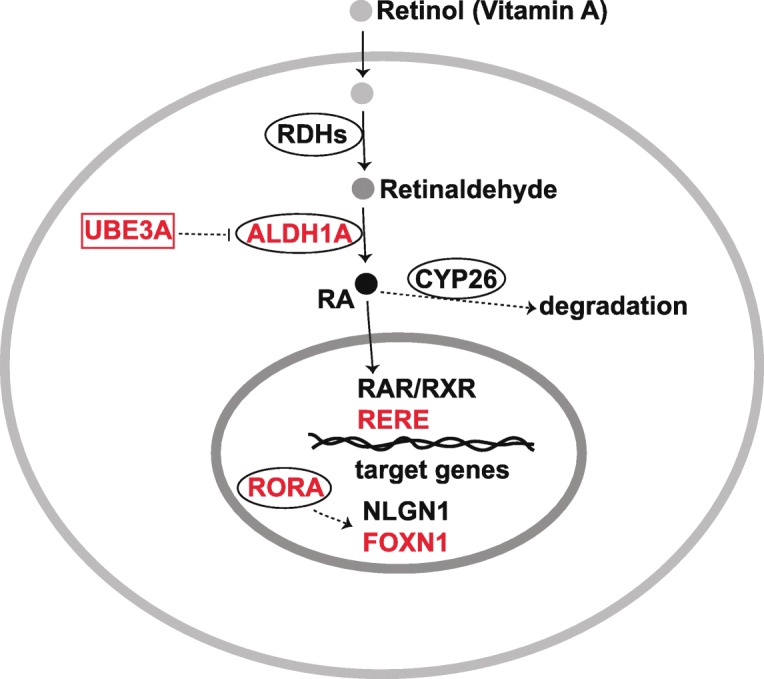
Possible interactions between ASD causal genes and RA signaling. UBE3A affects ALDH1A expression and thereby affects retinoic acid signaling pathway. RORA is associated with ASD which influences NLGN1. The RA signaling coregulator RERE is also associated with ASD

Retinoic acid (RA), the functional metabolite of vitamin A, is an essential morphogen in vertebrate development [126, 127]. RA mediates both genomic transcriptional effects by binding to nuclear receptors called retinoic acid receptors (RARs) and retinoid X receptors (RXRs) as well as non-genomic effects such as retinoylation (RA acylation), a post-translational modification of proteins [128, 129]. A range of co-activators and co-repressors have been reported in modulating RA signaling activity [129]. In the developing CNS, RA is required for neural patterning, differentiation, proliferation, and the establishment of neurotransmitter systems [130]. RA from the meninges regulates cortical neuron generation [131]. Vitamin A deficiency may induce ASD-like behaviors in rats [132]. It has been proposed that an abnormality in the interplay between retinoic acid and sex hormones may cause ASD [19]. Aberrant methylation and decreased protein expression of retinoic acid-related orphan receptor alpha (RORA) have been found in the autistic brain [133], while *RORA* variants have been associated with ASD [134]. Whole-exome sequencing in a South American cohort links RA signaling genes, including an RA-synthesizing gene aldehyde dehydrogenase 1 family member A3 (*ALDH1A3*) and the RORA-regulated *FOXN1* to ASD [135]. Low level of *ALDH1A1* has been found in a subset of autistic patients [136]. De novo mutations in arginine-glutamic acid dipeptide repeats (*RERE*) that encode a nuclear receptor coregulator for RA signaling may cause ASD and other defects associated with proximal 1p36 deletions [137]. Genome-wide chromatin immunoprecipitation analysis revealed that RORA transcriptionally regulates several ASD-relevant genes, including *NLGN1* [138]. Intriguingly, overexpression of UBE3A represses *ALDH1A2* and impairs RA-mediated synaptic plasticity in ASD, which can be alleviated by RA supplements [139]. All-*trans*-RA can upregulate the reduced CD38 expression in lymphoblastoid cell lines from ASD, while CD38-deficient mice exhibit ASD-like behavior [140, 141]. Beta-carotene, a precursor of vitamin A, has been shown as a potential treatment of autistic-like behavior in BTBR mice [142]. A synthetic RORA/G agonist has been tested to alleviate autistic disorders in a mouse model [143]. These studies suggest therapeutic approaches for treating ASD by targeting RA and related signaling pathways. The possible interactions among ASD causal genes and RA signaling have been described in Fig. 3.

## Non-genetic etiologies of ASD and altered signaling pathways

Various exogenous factors, such as prenatal exposure to viral infection or VPA, lead to several neurodevelopmental disorders with perturbed WNT signaling (Fig. 1). The transcription factor GATA-3 is critical for the brain development [144] and is involved in the WNT [145] and TGF-β/BMP [146, 147] signaling pathways. An increase in binding to GATA sites in DNA has been reported while exposure to thalidomide, valproate, and alcohol is known to cause ASD [148].

### Viral infection

The infection with rubella in early pregnancy has been linked to autism in clinical and epidemiological studies [149]. Prenatal viral-like immune activation has recently been reported to induce stable hyper- and hypomethylated CpGs at WNT signaling genomic regions (*WNT3*, *WNT7B*, *WNT8A*) which further disrupt the transcription of downstream target genes [150], suggesting a potential role of epigenetic modulation of WNT signaling in ASD etiologies.

### VPA

The use of the anticonvulsant valproic acid (VPA) during early pregnancy has been reported to cause autism in 11% children and autistic traits in an even larger number of children [151]. VPA is used in the treatment of epilepsy and bipolar disorder. However, rat prenatal exposure to VPA results in animals that are susceptible to autism-like phenotypes [20]. Prenatally VPA-treated rats exhibit an imbalance in oxidative homeostasis that facilitates susceptibility to autism [20, 152]. VPA treatment in rats resulted in lowered social interaction, longer moving time in the central area, and reduced standing times. Sulindac is a small molecule inhibitor of the WNT/β-catenin signaling pathway [20, 152]. Sulindac treatment can correct the VPA-induced autistic-like behaviors, p-Gsk3β downregulation, and β-catenin upregulation in the prefrontal lobe, hippocampus, and cerebellum [152].

## Conclusions

**Fig. 4:**
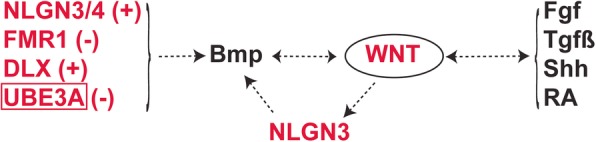
ASD causal genes affecting BMP signaling and potential crosstalk with other signaling pathways. ASD causal genes-encoded proteins, such as NLGN3/4, FMR1, DLX, and UBE3A, interact with BMP signaling pathway which may further affect WNT signaling. It should be noted that overexpression of UBE3A affects WNT and RA signaling pathways. However, its loss-of-function affects BMP signaling. Note: plus sign indicates upregulation; minus sign indicates downregulation

ASD causal genes may act upstream or downstream of WNT, BMP/TGFβ, SHH, FGF, and RA signaling pathways in vertebrates and invertebrates (Table 1 and Figs. 1, 2, 3, and 4). Alteration in these signaling pathways during brain development seems to cause ASD and other neurodevelopmental disorders. Previous studies support possible roles for these signaling pathways in the design of therapeutic targets for autism. However, systematic developmental studies are required to identify the temporal window in which impairment of these signals has the most significant impact on brain structure and function and resulting behavioral impairment. Such studies may also help in elucidating the upstream and downstream signaling pathways in the etiology of neurodevelopmental disorders as well as the mechanisms behind a particular impaired behavior. Although crosstalk among signaling pathways has been reported in several developmental processes and related diseases, similar studies in autistic models are lacking. Crosstalk between WNT and Hedgehog/Gli signaling in colon cancer has been studied and suggested as a potential target for its treatment [153]. β-Catenin and Gli1 are negatively regulated by GSK3β and CK1α [154–156] and have antagonistic roles in regulating TCF and downstream target genes in metastatic colon cancer [157]. The suppressor of the fused kinase (Sufu), a negative regulator of Gli1, has been reported to regulate the distribution of β-catenin in the nucleus and cytoplasm [158–160]. In colon cancer, loss of either PTEN or p53 leads to the activation of both β-catenin and Gli1 [157, 161]. The inhibition of SMO, an upstream active factor of Gli1, has been shown to reduce active β-catenin levels and induce its nuclear exclusion [162]. Gli1 negatively regulates Gli3R and vice versa [157]. Further, Gli3R has been shown to inhibit the activity of β-catenin [163]. The transcription of *Wnt2b*, *Wnt4*, and *Wnt7b* is shown to be induced by Gli1 [164]. The crosstalk between TGF-β and SHH pathways has also been reported in cancer [165], as well as in cyclosporine-enhanced cell proliferation in human gingival fibroblasts [166]. Neuropilin-1 (NRP1), a TGF-β co-receptor expressed on the membrane of cancer cells, is known to enhance the canonical SMAD2/3 signaling in response to TGF-β [167]. Further, HH signaling increases NRP1 transcription and NRP1 is also reported to increase the activation of HH target genes by mediating HH transduction between activated SMO and SUFU [168, 169]. While TGF-β is important for SMO-mediated cancer development [170], its role in the induction of GLI2 and GLI1 expression by inhibition of PKA activity has also been reported [171]. A hierarchical pattern of crosstalk has been suggested in which TGF-β upregulates Shh and leads to cyclosporine-enhanced Shh expression and cell proliferation in gingival fibroblasts [166]. Crosstalk between FGF and WNT pathways has been observed in zebrafish tailbud [172] and mouse craniofacial development [173]. Reciprocal positive regulation between FGF and WNT signaling has been observed [172]. WNT/β-catenin signaling in the anterior neural ridge and facial ectoderm has been shown to positively target Fgf8, and β-catenin GOF leads to ectopic expression of Fgf8 in the facial ectoderm [173]. Wnt has been reported to increase FGF signaling within the Mapk branch by elevating Erk phosphorylation levels [172]. Further, Fgf has been shown to inhibit the Wnt antagonists, dkk1 and notum1a, resulting in the elevation of WNT signaling [172]. A LOF mutation in *UBE3A*, an ASD-associated gene, influences both the WNT and BMP signaling pathways, suggesting possible crosstalk between them [12, 78]. Xu et al. further demonstrated that excessive *UBE3A* impairs RA-mediated neuronal synaptic plasticity in ASD probably by negative regulation of ALDH1A2, the rate-limiting enzyme of retinoic acid (RA) synthesis. [139]. Medina et al. [81] suggested that while *Nlgn3* is a direct target of WNT/β-catenin signaling, the ASD-associated gene may also regulate BMP signaling. These results suggest that signaling crosstalk among morphogenetic pathways is mediated by autistic causal genes, thereby demonstrating value in further in-depth studies on interactions between signaling molecules in normal physiological and diseased conditions. Evidence has suggested a tissue-specific mechanism behind WNT and BMP signaling crosstalk [174]. Moreover, WNT signaling may repress RA signaling during orofacial development [175], while WNT signaling positively regulates RA signaling in the dorsal optic cup during eye development [176], suggesting context-dependent mechanisms of signaling interactions. Therefore, the interaction between various signaling pathways should be studied in neuronal as well as glial cells for ASD, which may help in designing treatment and targeting perturbed signaling in a cell-specific manner. Among the nine high-confidence ASD risk genes, only a few have been studied so far in the context of impaired signaling pathways. The investigation of roles for other ASD genes in neurodevelopment and in the regulation of various signaling pathways may increase the understanding of mechanisms behind the etiology of ASD. Overall, this article proposes to study how different ASD causal genes interact with each signaling pathway in the development of the brain and whether there is any crosstalk between them.

## Abbreviations

AIS: Axon initial segment
ALDH1A3: Aldehyde dehydrogenase 1 family member A3
ANK3: Ankyrin-G
APC: Adenomatous polyposis coli
ASD: Autism spectrum disorder
BDNF: Brain-derived neurotrophic factor
BMP: Bone morphogenetic protein
Bmper: BMP binding endothelial regulator
BMPR2: Bone morphogenetic protein type II receptor
CHD8: Chromodomain helicase DNA-binding protein 8
cKO: Conditional knockout
CTNNB1: β-Catenin
DHCR7: 7-Dehydrocholesterol reductase
DIXDC1: DIX domain containing 1
DNlg4: Drosophila neuroligin 4
DNVs: De novo variants
EN2: Engrailed2
FGF: Fibroblast growth factor
FMR1: Fragile X mental retardation 1
FMRP: FMR1 protein
FXS: Fragile X syndrome
GOF: Gain-of-function
hiPSC: Human induced pluripotent stem cell
IHH: Indian hedgehog
LIMK1: LIM domain kinase 1
LOF: Loss-of-function
mGluR5: Metabotropic glutamate receptor 5
NAc: Nucleus accumbens
NGF: Nerve growth factor
NGSEA: Network-based gene set enrichment analysis
NLGN: Neuroligins
Nlgn3: Neuroligin 3
NMJs: Neuromuscular junctions
OFR: Oxygen free radicals
PCP: Planar cell polarity
PGE2: Prostaglandin E2
PTCHD1: Patched domain-containing 1
RA: Retinoic acid
RERE: Arginine-glutamic acid dipeptide repeats
RORA: Retinoic acid-related orphan receptor alpha
SHH: Sonic hedgehog
SMO: Transmembrane protein Smoothened
TGF-β: Transforming growth factor β
Tkv: BMP type I receptor thickvein
UBE3A: Ubiquitin-protein ligase E3A
VPA: Valproic acid
